# Exploring drivers and prevention strategies for sexual violence among adolescent girls and young women in Kicukiro, Rwanda

**DOI:** 10.3389/frph.2025.1420947

**Published:** 2025-01-29

**Authors:** Wongani Patricia Kawonga, Sam M. Livingstone, Augustine Ndaimani, Jean Pierre Sibomana, Tsion Yohannes Waka, Maxwell Mhlanga, Andrew Mclellan

**Affiliations:** ^1^ Center for Gender Equity, University of Global Health Equity, Kigali, Rwanda; ^2^ Center for Nursing and Midwifery, University of Global Health Equity, Kigali, Rwanda; ^3^ Administration, Young Women’s Christian Association of Rwanda, Kigali, Rwanda; ^4^ Faculty of Nursing, Muhimbili University of Health and Allied Sciences, Dar es Salaam, Tanzania

**Keywords:** adolescent girls and young women (AGYW), drivers, Kicukiro, prevention strategies, Rwanda, sexual violence, Young Women's Christian Fellowship

## Abstract

**Introduction:**

Sexual violence against adolescent girls and young women (AGYW) is a pervasive issue globally, with particularly high prevalence in sub-Saharan Africa. This study explores the drivers and prevention strategies for sexual violence among AGYW in Kicukiro, Rwanda.

**Methods:**

A descriptive exploratory design guided by the socioecological model and feminist standpoint theory was employed. In-depth interviews were conducted with 13 AGYW participating in a mentorship program and 5 male community members. Data was analyzed using thematic analysis to identify key drivers and potential prevention strategies.

**Results:**

Four themes emerged: (1) empowering mentorship programs, (2) tiered drivers of sexual violence, (3) optimizing violence-mitigating resources, and (4) interference with judicial processes. Key drivers of sexual violence included substance use, transactional sexual relationships, inadequate family protection, entrenched gender stereotypes, and limited legal literacy.

**Conclusion:**

Findings underscore the need for multifaceted interventions that address systemic and cultural barriers, strengthen legal frameworks, and expand community-based programs. Addressing sexual violence requires a holistic approach, integrating empowerment initiatives, robust community engagement, and legal reforms to create safer environments for AGYW. The mentorship program's success suggests scalability across other settings.

## Introduction

The global prevalence of sexual violence continues to escalate despite ongoing efforts to address this pervasive issue. Notably, low-resource settings bear a disproportionately high burden of this problem (1). Worldwide statistics reveal that approximately one in three (35%) women and girls experience either intimate partner violence or non-partner sexual violence during their lifetime. Moreover, almost one in four (24%) young women (aged 15–24 years) undergoes sexual violence by the time they reach their mid-20s (1).

In sub-Saharan Africa, the figures are alarming, with 20% of women experiencing both non-intimate and intimate partner sexual violence in their lifetime and a staggering 45% of adolescent girls reporting forced sexual experiences (1). In Rwanda, sexual violence remains a widespread concern, with documented cases rising from 12.9% in 2005 to 22.4% in 2014–2015 and persisting at 22.5%, according to the latest available data from DHS 2019–2020. Notably, Rwanda annually spends around US$ 1.5 million to manage complications arising from unsafe abortions among teenagers, predominantly linked to incidents of sexual violence (2). A recent report showed that 56% of rape convictions involved girls below the age of 15, emphasising the particularly vulnerable position of young individuals in the face of sexual violence (3). These disconcerting figures underscore the urgent need for comprehensive and sustained efforts to address sexual violence on both a global and localised scale.

Sexual violence refers to any non-consensual sexual activity inflicted upon an individual, whether due to lack of consent or the inability to provide consent (1, 4). These situations can arise when the person involved is underage, mentally incapacitated, rendered unconscious, or severely impaired by drugs or alcohol. Sexual violence manifests in various forms, including rape, forced nudity, forced abortion, and harassment (5). It occurs both within intimate relationships and outside of them. Intimate sexual violence includes rape or sexual assault, child sexual assault and incest, and intimate partner sexual assault. On the other hand, non-intimate partner sexual violence encompasses unwanted sexual contact or touching, sexual harassment, sexual exploitation, such as exposing one's genitals or naked body to other people without their consent, and voyeurism, which involves observing someone engaged in a private act without their knowledge or permission (6).

Sexual violence has far-reaching and devastating impacts on individuals and communities. It can lead to fatal outcomes and cause physical injuries. Additionally, it is linked to unintended pregnancies, induced abortions, gynaecological issues, and sexually transmitted infections, including HIV. The psychological toll is profound, leading to depression, post-traumatic stress disorder, anxiety disorders, sleep difficulties, and eating disorders. Disturbingly, survivors of childhood sexual violence may develop harmful coping mechanisms, such as increased smoking, substance use, and risky sexual behaviours (1). Eliminating violence is essential for achieving a society where everyone can live with dignity, security, and opportunity (7).

Despite Rwanda having a robust legal framework for addressing sexual violence against women and having civil society organisations complement government efforts to promote women's empowerment and violence prevention, sexual violence persists. There has been growing research on sexual violence in Rwanda. Still, there is limited evidence or information on the drivers of sexual violence against adolescent girls and young women (AGYW) in the Kicukiro district. To the best of our knowledge, no previous study has investigated the factors driving sexual violence against AGYW in Kicukiro district from the perspectives of AGYW and men. While some studies conducted in Rwanda have focused on addressing gender norms (8), identifying the root causes of sexual gender-based violence (GBV) (9), and trends of intimate partner violence (10), most of them are nationwide cross-sectional studies and surveys, lacking a specific emphasis on the perspectives, beliefs, experiences, and effective solutions as perceived by AGYW in their unique context. The Young Women's Christian Association (YWCA) conducts a mentoring project on sexual and reproductive health and rights (SRHR) in Kicukiro district. The organisation was concerned about the prevalence of sexual violence and wanted further to understand its drivers as part of its program evaluation. This study sought to identify the drivers of sexual violence against AGYW, specifically exploring individual, relationship, community, and societal drivers of sexual violence against AGYW to identify strategies and challenges for prevention.

## Materials and methods

### Study setting

This project was carried out in partnership with the Young Women's Christian Association (YWCA) in Kicukiro district, one of Rwanda's 30 districts. The district is in the southeast of Kigali city and was chosen for the study due to its high prevalence of HIV, teen pregnancy, and sexual violence among AGYW (11, 12). Kicukiro District has a total population of 491,731, with 249,115 males and 242,616 females, 58.1% of whom are younger than 25 (11, 12). This study focused on the 15–24 age group, constituting 14.2% of the population in the district. This study was conducted in three sectors of Kicukiro, namely Gahanga, Niboye and Masaka, where the YWCA conducts the SRHR mentorship program, which targets AGYW and boys.

YWCA operates in 15 districts in all the Provinces of Rwanda to develop the leadership and collective power of girls and women in Rwanda, enabling them to achieve high-quality education, health, and socio-economic. Since 2018, the YWCA SRHR Mentorship Program has targeted AGYW with age-appropriate interventions and capacity-building modules. The mentorship program, peers and field offices provide AGYW with comprehensive training on reproduction, reproductive health and rights, and prevention of gender-based and sexual violence, utilising safe space modules such as “Grow Up Smart” and “Power to Change.” These modules are intended to improve AGYW with assertiveness and support healthy relationships with their families and communities, with the hopes of reducing their vulnerability to sexual violence, unwanted pregnancies, sexually transmitted infections, and substance use (13). Program participants are expected to attend one weekly session for 1 year, after which they graduate and may become peer mentors. Furthermore, the program includes the provision of menstrual hygiene products and the further training of peer mentors.

### Methodology

A descriptive exploratory study design was employed, utilising in-depth interviews to explore, document, and characterise the perspectives of AGYW within the SRHR mentorship program, their male relatives, and/or men in local leadership roles on the drivers of sexual violence against AGYW. The design allowed pattern recognition and integration with existing knowledge and theories (14). Sexual assault is a complex problem involving multiple interacting variables and influences from survivors, perpetrators, families, communities and policy. Therefore, the study was guided by the socioecological model (SEM). The SEM is effective in analysing complex human events such as sexual assault (15). According to the SEM, the multidimensional influences of human behaviour include individual, interpersonal, community and societal domains (15). This study additionally incorporated the feminist standpoint framework, where violence is characterised by power imbalances which disadvantage women and their gender (16). The feminist standpoint theory puts forth three foundational claims. First and foremost is the assertion that knowledge is socially situated, acknowledging that our understanding of the world is shaped by the specific social context in which it is produced. Secondly, marginalised groups occupy distinctive social positions, affording them a heightened awareness of their lived experiences. Lastly, the feminist standpoint theory emphasises the capacity of marginalised individuals to pose relevant questions about their circumstances, particularly concerning power dynamics. This claim underscores the importance of commencing inquiries into power relations by focusing on the lives and perspectives of the marginalised in society. Its significance lies in its ability to facilitate the deconstruction and understanding of power dynamics and oppression, emphasising the importance of prioritising the perspectives of marginalised individuals to gain a more nuanced understanding of power structures and the impact of oppression (16, 17). Together, these two theories worked synergistically to guide the exploratory inquiry by offering complementary lenses to understand and interpret the drivers of sexual violence in the studied context.

### Sample

A heterogeneous purposive sampling technique was employed in the recruitment of participants. Thirteen Adolescent Girls and Young Women (AGYW) from the Sexual and Reproductive Health and Rights (SRHR) mentorship program and five male relatives of the AGYW who held community leadership positions were included. The AGYW sample encompassed both in-school (primary and secondary) and out-of-school individuals, comprising survivors of sexual violence and those who had not experienced such incidents.

To be eligible for inclusion, AGYW were required to be between the ages of 15 and 24, reside in Kicukiro, and graduate from the one-year SRHR mentorship program. Males were included if they lived or worked in Kicukiro and were either related to the included AGYW or held community leadership roles. The inclusion of males aimed to provide a broader understanding of the drivers of sexual violence by capturing perspectives from individuals who may influence or be influenced by the societal and relational dynamics surrounding AGYW. Their participation contributed to the triangulation of data, enriching the analysis by incorporating viewpoints from those who occupy influential social or familial roles. AGYW currently experiencing violence, undergoing court processes, or unable to provide assent and/or informed consent were excluded from the study. Similarly, males who could not give consent were also excluded from participation. This sampling strategy aimed to ensure a diverse and representative participant group while respecting ethical considerations and safeguarding the well-being of potential participants.

### Data collection and management

The data collection employed a vernacular semi-structured interview schedule, covering demographic information, experiences, perceived drivers of sexual violence, strategies, challenges at various levels, and opportunities for the prevention of sexual violence towards AGYW. The interview guide comprised eight questions, including one for rapport-building, a transition question, five main questions, and a closing question, all with relevant probes. The tool underwent pretesting with three postgraduate Global Health students fluent in both English and Kinyarwanda languages and six additional individuals who were either SRHR mentees or YWCA officers who did not eventually participate in the study. Pretesting was conducted to refine the interview guide by ensuring its clarity, cultural relevance, and appropriateness for the target population. This process also helped identify potential issues with question phrasing or structure, ensuring that the tool effectively captured the intended data while minimising ambiguity or discomfort for participants.

Ethical approval for the study was obtained from the University of Global Health Equity (UGHE) institutional review board (Ref: UGHE-IRB/2023/0233), and voluntary consent was sought from all participants above the age of 18 years. As this study was part of an evaluation exercise for the SRHR mentorship program, which included minors, four participants below 18 were included in the sample; in these cases, informed consent to participate in the study was granted by the individual AGYM and their parents. The consent form and the questionnaire were expressed in simple language. All five minors in this study were in secondary school. Minors were accompanied by a YWCA Field Supervisor and their parents during the interview with the research assistants. The interviews took place in a private room at the Masaka Sector office. Other interviews were conducted at a Hospital in Niboye and a school in Gahanga. Participants were informed that the interviews would be audio-recorded and that they had the right to accept or decline to be recorded. One participant refused to be recorded and subsequently cancelled the interview. YWCA Field officers checked on the participants a week after the study to see if the interviews had negatively affected them. However, no reported emotional or physical harm occurred during the study. Principles of Good Clinical Practice, as detailed in the Belmont Report and the Declaration of Helsinki, guided the conduct of this study. Additionally, the research team adhered to Rwandan legal obligations, ensuring that any disclosure of rape involving minors would be reported to the appropriate authorities while maintaining participant confidentiality and dignity under the oversight of an Institutional Review Board (IRB).

The YWCA identified and recruited participants from the SRHR mentorship program database. Interviews were conducted in person at locations convenient to the participants in Niboye, Masaka, and Gahanga and recorded using an Aiworth® digital voice recorder. Confidentiality was maintained throughout the study, with identifiable information kept separately. Unique identifiers protected participant identity. Interviews lasted 20–40 min and were transcribed verbatim in Kinyarwanda before translation into English.

Demographic information was entered into Dedoose software, accessible only to the research team, and analysed to create a general profile of study participants. Audio recordings were uploaded to password-protected computers, only accessible to the research team and data collectors. Audio files were deleted from recorders, and all data, including transcripts and field notes, were stored securely. Consent forms, field notes, and personal information were coded, safeguarded, and stored in a locked cabinet. Data will be retained at UGHE for 10 years post-study completion before being destroyed.

### Data analysis

This study ascertained data saturation through two primary methods: coding during data collection and feedback obtained during field meeting briefings. The data collection, transcription, and translation process occurred concurrently, with the team incorporating two days of breaks between data collection sessions. This interval allowed the team to review and identify patterns within the collected data thoroughly.

Coding was done collaboratively by WPK, SML, and AN. Before coding and data analysis, these team members documented their positionality and kept analytic memos in the audit trail, ensuring transparency and addressing personal biases. Each research team member conducted an open reading of the 18 transcripts to immerse themselves in the content, identify emerging concepts, and develop a preliminary codebook based on interview responses. Subsequently, the research team convened to consolidate the code structure.

Based on the established codebook, all transcripts were subjected to deductive and iterative coding using manual techniques and the Dedoose software. The data underwent both deductive using the ecological framework and inductive thematic analysis, with themes emerging from the data identified and explored by team members, first individually and then collaboratively. To enhance the study's trustworthiness, the research team implemented member checking and engaged in peer debriefing processes. These measures aimed to validate the findings and ensure the reliability and credibility of the study outcomes.

## Results

### Demographics

There were eighteen participants: seven (38.9%) each from Niboye and Gahanga and four (22.2%) from Masaka. Five participants were males, two community leaders, and three AGYW partners. Four AGYW (22.5%) were aged 15–17 years, three (16.7%) aged 18–20 years and six (33.3%) aged 20–24 years. Eight (44.4%) participants were still students, while the rest were in small-scale business or other menial jobs. The demographic profile of participants is summarised in Table 1.

**Table 1:** Socio-demographic characteristics of the participants.

Participant ID	Age	Sex assigned at birth	Marital status	School going	Level of education	Source of financial support	Place of residence
PID 001	21	Female	Single	Yes	Secondary 3	Parents	High-density suburb
PID 002	18	Female	Single	Yes	Secondary 4	Parents	High-density suburb
PID 003	24	Female	Single	Yes	Primary 4	Self-employed	High-density suburb
PID 004	24	Female	Single	Yes	Secondary 6	Self-employed	High-density suburb
PID 005	24	Male	Single	No	Primary 3	Self-employed	High-density suburb
PID 006	20	Male	Single	No	Primary 4	Parents	High-density suburb
PID 007	24	Female	Married	No	Secondary Graduate	Self-employed	High-density suburb
PID 008	26	Male	Married	Yes	University Undergraduate	Employed	High-density suburb
PID 009	16	Female	Single	Yes	Secondary 4	Parents	High-density suburb
PID 010	21	Female	Single	No	Secondary 3	Employed	High-density suburb
PID 011	15	Female	Single	Yes	Secondary 3	Parents	High-density suburb
PID 012	30	Male	Married	No	University Undergraduate	Self-employed	High-density suburb
PID 013	17	Female	Single	Yes	Secondary 3	Parents	High-density suburb
PID 014	23	Female	Single	No	Secondary graduate	Parents	High-density suburb
PID 015	22	Female	Single	Yes	Primary 6	Self-employed	High-density suburb
PID 016	17	Female	Single	Yes	Secondary 3	Parents	High-density suburb
PID 017	28	Male	Single	No	Secondary 4	Employed	High-density suburb
PID 018	18	Female	Single	No	Primary 4	Parents	High-density suburb

### Themes

Four themes emerged, namely (1) empowering SRHR mentorship, (2) tiered drivers of sexual violence, (3) optimising existing violence mitigating structures and (4) interference with judicial processes, Table 2.

**Table 2:** Themes and subthemes.

Theme	Subtheme	Code
Empowering SRHR mentorship	Access to services, support, and SRHR knowledge	• Training on reproductive health • Knowledge of prevention of gender-based violence • Access to ^a^SRHR services • Skills to prevent sexual abuse
	Economic and livelihood empowerment	• Acquisition of entrepreneurial and budgeting skills • Personal and social empowerment
	Safe spaces for sharing experiences and building confidence	• Psychological safety at ^b^YWCA sites • Discussion in private spaces • Counselling and support services • Peer mentorship and support
Tiered drivers of sexual violence	Practices increasing vulnerability	• Dressing perceived as sexually suggestive • Recreational drug use by victims • Recreational drug use offenders • Hanging out in areas where sexual abuse abounds
	Transactional sexual relationships	• Poverty resulting in sex for reward • Peer pressure to engage in sexual relations • Materialism • Social pressure • Economic vulnerability
	Inadequate family protection	• Absence of parents • Intergenerational abuse • Low-income family relationships • Neglected children as unintended consequences of empowerment • Family conflict • Protecting family reputation through silence • Some young girls resist parental advice
	Gender stereotypes and misconceptions	• Harmful masculine norms • Religious justification of male dominance • Gender bias suppressing the needs of girls and young women • Misconceptions about menses • Power gradient in favour of males • Beliefs about the superiority of males • Economic disparities • Manipulation of victims by offenders • Justification of sexual violence
	Poor legal literacy	• Lack of knowledge about sexual and reproductive health rights • Lack of awareness of supportive services
Optimising existing violence-mitigating structures	Comprehensive SRHR education and support	• Safe SRHR discussion platforms • Fighting drug and alcohol abuse • The existing SRHR mentorship program
	Family and parental support	• Involvement of fathers • Involvement of mothers • Open communication lines in the family
	Community support	• Need to expand coverage of the SRHR program • Committed community leadership • Implementing community-driven solutions
	Economic empowerment for ^c^AGYW	• Engaging youths in productive activities • Supporting the education of girls and young women
	Strengthening legal frameworks and practices	• Existing laws and structures for SRHR • Existing parents' evening session on ^d^GBV • Stricter penalties for perpetrators • Integrating SRHR services • Victim friendly services
Interference with judicial processes	Family concealment of sexual assault	• Selective reporting of sexual abuse • Lack of reporting by community leaders • Bribing community leaders • Fear of negative portrayal of the community fabric • Stigma and fear of negative perception
	Complicity in reporting and lack of confidentiality	• Reporting involves unprofessional community leaders who may further tarnish the victim’s image • Litigation process violates privacy and confidentiality
	Bribery and lengthy litigation processes	• Corrupt tendencies among some judicial officials • Lengthy reporting and judicial processes for sexual assault • Reporting barriers due to the involvement of community leaders

^a^ SRHR, sexual and reproductive health and rights.

^b^ YWCA, Young Women’s Christian Association.

^c^ AGYW, adolescent girls and young women.

^d^ GBV, gender-based violence.

Participants expressed how the SRHR mentorship program improved their access to services, support, financial security and SRHR knowledge. For instance, one participant showed that the program provided her with a safe space to share experiences, express her concerns, build self-confidence and make informed decisions about her sexual and reproductive health.

We learnt to share our stories of sexual harassment where we get support from mentors and supervisors… they taught us how to protect ourselves from sexual offences from peers and close friends and any other form of abuse and know how to identify someone who would abuse you or start reporting when you know if someone has been abused. (Female, 19 years old, PID 001)

Moreover, participants noted the program's impact on livelihood and economic empowerment. One participant highlighted her newfound confidence in rejecting advances for material gifts, emphasising the importance of earning and saving money independently, “If a man will want to lure me into having sex with me for a gift, I am confident to tell him I get what I want by working for it and saving money” (Female, 21 years old, PID 010).

In addition to personal and economic empowerment, participants acquired crucial SRHR knowledge and skills through the program. A participant shared her insights on sexual violence prevention and emphasised the program's guidance on seeking care from nearby health centres. Another participant noted she “gained significant knowledge about sexual violence prevention and we are also advised to get care from the nearest health centres.” (Female, 18years, PID 002). Other knowledge gained from the mentorship program included “how a girl goes on her period, how she behaves. importance of standing by my No or Yes decision” (Female, 19years old PID 001).

### Tiered drivers of sexual violence

Drivers of sexual violence were tiered along personal, interpersonal, community and societal levels. The drivers included practices perceived to increase vulnerability to sexual violence, limited family protection, gender stereotypes and poor legal literacy.

#### Practices perceived to increase vulnerability

Participants pointed out that vulnerability to sexual assault increased when AGYW were under the influence of alcohol or recreational drugs and putting on dressing which perpetrators perceived as sexually suggestive. Participants said, “individuals who experience sexual abuse are either present in alcohol-drinking establishments or are consuming alcohol themselves” (Male, 30 years, PID 012*)* and “When the girls drink the alcohol, they would either fall asleep or become vulnerable to manipulation and rape” (Female, 15 years old, PID 011). Another participant also concurred that*,* after taking alcohol or drugs, girls “can easily be harassed, sexually abused… men take advantage” (Female, 23 years old, PID 014). On the other hand, men who abuse alcohol “are more likely to be involved in acts of sexual harassment and rape against their spouses or young girls” (Male, 30 years, PID 012*).* Regarding dressing, “when men look at them in short clothes, they wish to sleep with them and target them for rape” (Female, 17 years old, PID 016). One participant (Female, 23 years old, PID 014) felt that AGYW may need to avoid sexual assault hotspots when putting on elegant dressing.

Transactional sexual relations were recognised as another driver of sexual violence at the interpersonal level, as some impoverished girls and young women tried to meet their needs in the process. One young woman said, “you yearn for things you don't possess, and men offer them to you in exchange for sex” (Female, 24 years, PID003), compromising girls' decision-making and bodily autonomy and exposing them to sexual exploitation and assault.

Poverty leads people to drugs and alcohol as a way of coping, which drives them into violence. There are four [orphaned] children …, they started engaging in alcohol and drug abuse … the girl ended up being pregnant, while the boy ended up in prison. (Female, 24 years old, PID004)

The sexual transaction became complete: “as a girl who wants her needs met, I end up giving him sex in exchange for money and things I want” (Female, 21 years old, PID 010). Some girls felt the need to pay for fine living through transactional sex as “no man can give you money for buying a phone without sex.” (Female, 24 years old, PID 004). Such transactions are not easily made public. The man “gives her a job in exchange for sex and impregnates her and gives her money to remain quiet.” (Male, 24 years, PID 005). Girls and young women felt powerless,

“when a boy is coming from a rich family, and he finds a girl from a … poor family, … when he decides to sleep with you, there is nothing you can do about it” (Female, 18 years old, PID 002)

#### Inadequate family protection

The absence of adequate family protection mechanisms, including parental absence due to work or conflicts, was identified as a driver of sexual violence at the community level. Some participants said girls were usually left alone at home as their parents pursued income-generating activities. As a result, some girls are assaulted without their parent's knowledge while perpetrators remain free to commit other sexual assault crimes, promoting the cycle of violence to continue. One girl “used to sleep with men because her mother used to leave her alone at home and go to work and come back late” (Male, 20 years old, PID 006). One participant felt that “the root cause of violence is mainly caused by parents’ absence in the lives of their children … which leaves children with no chance or time to talk to their parents” (Female, 23 years old, PID 014).

Furthermore, “when your parents are not on good terms, they can also cause violence” (Female, 16 years old, PID 009). In fact, “family conflicts create a hostile and unstable environment where tensions escalate, leading to an increased risk of sexual and physical violence due to a lack of emotional support, attention to children, and broken communication” (Female, 21 years old, PID 010).

In addition, sexual assault by family, friends, neighbours, and relatives usually goes unreported to protect family relations.

The man sexually abused her more than four times. The child said that it was her alcoholic father-in-law who sexually abused her. The mother said it did not matter since this was her husband. (Female, 24 years old, PID 007)

When concealing the sexual assault, “your mother may know about this, but she tells you never to tell anyone about the issue.” (Female, 15 years old, PID 011). The concealment was done to give the perpetrators a chance to fend for the assaulted girl.

The family of the boy who impregnated her comes to visit your family and tells them that if they report this, we will not be helping. Your family agrees to remain quiet to get help and protect the perpetrator's reputation. (Female, 16 years old, PID 009)

#### Gender stereotypes and misconceptions

Participants revealed that male dominance, superiority, control, and female subordination were quite common in their neighbourhood. Some men “believe that a man is superior and that their word is final” (Female, 18 years old, PID 002) and “women are like objects that they can use in any way they want.” (Male, 30 years, PID 012). Misconceptions about menses resulted in some girls getting tricked into attempting to treat severe menstrual symptoms through sexual intercourse.

Some girls always have abdominal pain during their menses, and men will tell them that when you give birth, that's when you will be fine, or tell them that when I sleep with you, you will be fine, and because they want to be healed, they will accept. (Female, 21 years old, PID 010)

#### Poor legal literacy

Participants expressed concerns about the limited knowledge about sexual violence, including its forms, impacts, what is reportable and available support services. The public may not know what forms of violence they can deal with as a family and which forms of violence should be reported. Participants indicated that this lack of information contributes to a culture of silence, making it difficult for survivors to seek help or report incidents. Additionally, “many people, … don't understand what sexual violence is, … what the law says … because of this, they can't do anything or change anything, even if violence happens.” *(*female, 23 years old, PID 014).

Most adolescent girls affected by sexual violence do not have enough information about sexual violence and its forms… As a result, an older person might … coerce them into … sex against their will, and they may feel obliged to comply due to their cultural values. (Male, 26 years old, PID 008)

### Optimising local violence mitigating resources

Participants emphasised the importance of a balanced, comprehensive, collective, and collaborative approach to reproductive health education within the family unit and the community. They identified key strategies and areas for improvement to mitigate sexual violence. Furthermore, participants stressed the need for comprehensive sexual and reproductive health (SRHR) education for both AGYW and their parents. This includes increasing awareness, addressing alcohol and drug abuse, and enhancing the effectiveness of protective laws. “Additionally, both parents need to have a conversation with their daughters about how men can deceive or manipulate them and teach them how to overcome such situations” (Female, 16 yrs., PID 009).

Participants pointed out that “many times, you find that it's only mothers who discuss with their children about reproduction, but fathers must” (Female, 16 years old, PID 009). Parents were encouraged to socialise their children into resilience “by instilling a sense of satisfaction during their early years, parents can help their children develop resilience against peer pressure and reduce their vulnerability to sexual violence as they grow up” (Female, 19 years PID 001).

Efforts to prevent sexual violence were recommended to involve victims, AGYW, families, and communities. Participants felt the need to “initiate campaigns within our families and communities to address … sexual violence… educate us on strategies to combat violence.” (Female, 21 years, PID 010).

Participants highlighted the importance of fair laws and practices already in place to prevent sexual violence. They mentioned the *Ikirambi* practice, a private discussion of intimate issues with trusted relatives, as a positive initiative that could lead to “a substantial reduction in all forms of violence against girls and women.” (Female, 24 years old, PID 007).

Public awareness of relevant laws for sexual assault and the channels for assistance was seen as crucial. Participants wanted to see justice being served and perpetrators punished for their actions against AGYW through “reliable and accountable authority or leaders, … to ensure that those who have committed violence against children are not spared from punishment.” (Female, 19 years, PID 001).

Participants called for “severe punishments for rapists and those that harass and sexually abuse girls to serve as a deterrent and sets an example for others so that it should not continue” (Female, 18 years, PID 002).

Economic empowerment of AGYW, particularly school dropouts, was highlighted as a strategy to reduce vulnerability. Participants believed that “if girls are employed where they can get paid at the end of every month or have a constant source of finance, I think violence can also be reduced.” (Male, 30 years PID 012).

These suggestions underscore the importance of a holistic and community-driven approach to addressing sexual violence, encompassing education, family involvement, community campaigns, legal frameworks, and economic empowerment.

### Interference with judicial processes

Addressing sexual violence against AGYW faces significant challenges due to interference with judicial processes. Several factors contribute to the difficulty in seeking legal recourse, perpetuating a cycle of abuse:

#### Concealment within families

Families may choose to conceal incidents of sexual assault, either out of fear, stigma, or a desire to protect their reputation. When the abused girl “goes there and confronts the mother of the boy who molested you. They both say let's keep quiet so as not to harm our family” (Female, 15 years old, PID 011). Ironically, the girl's family would also “keep this secretly because it is a scandal, and it will look bad for us” (Female, 21 years, PID 010).

#### Stigma and fear of negative perception

The stigma associated with sexual assault may lead families and “many leaders tend to conceal information about sexual violence, rape, and girls’ pregnancies in their villages, fearing that it may negatively portray their community to others” (Male, 26 years, PID 008) contributing to the underreporting of sexual violence.

#### Lack of confidentiality and perceived complicity

Reporting mechanisms often lack confidentiality, with leaders in the reporting line perceived as untrustworthy or complicit. “When you call the village leaders, and they ignore you, even the sector cannot listen to you because you cannot take yourself to the sector without going to the village first.” (Female, 18 years old, PID 018). Hence, there is a need to “establish an easy, accessible, and victim-friendly reporting” (Male, 26 years old, PID 008).

#### Bribery and lengthy litigation processes

Victims may be discouraged from pursuing legal recourse due to bribery and lengthy litigation processes. In some cases, “the perpetrator who raped her had successfully bribed the village or sector leaders so that he can't be arrested*.*” (Female, 21 years old, PID 010). Offenders who can afford to bribe authorities may escape justice, undermining the effectiveness of legal interventions.

[They] bribe the authority in charge of your case. They release him after being arrested, and he says that I won't do it again, but he does it again and again, knowing that he will give them money and they will release him. (Female, 15 years old, PID 011)

Efforts to address sexual violence must confront these challenges, promoting transparency, victim-friendly reporting mechanisms, and stringent measures against bribery to ensure justice prevails and break the cycle of abuse.

## Discussion

### The empowering impact of SRHR mentorship

This adds to the literature on sexual violence among AGYW from a feminist standpoint (18–22). Themes from the study showed institutionalised drivers of sexual violence at individual, interpersonal, community and societal levels, indicating that sexual violence needs to be addressed at all levels to achieve a lasting impact. The findings have shown that society tends to disenfranchise young girls and women against their male counterparts, thereby exposing them to sexual abuse, which, in turn, worsens their standing in society by worsening vulnerability and intersectional abuse. The SRHR mentorship program conducted by YWCA emerged as a crucial factor in transforming the lives of AGYW in the Kicukiro district. Participants reported improved SRHR knowledge, skills, economic empowerment, and well-being. This aligns with existing literature emphasising the positive impact of empowerment programs on women's lives, including protection against abuse and enhancement of autonomy (23–25). In addition, mentorship has been shown to facilitate the development of confidence, supportive relationships, and other soft and technical skills to deal with threats or violations (26). The mentorship program also provided safe spaces to share experiences. Apart from being assured (24), mentorship can also increase AGYW's social capital and autonomy over their health (27). This is in line with the feminist standpoint to improve the agency of AGYW to speak up, get support and challenge norms which perpetuate sexual violence. Kicukiro has been shown to have the highest poverty levels compared to other districts in Kigali city (28).

### Drivers of sexual violence

#### Practices perceived to increase vulnerability to sexual violence

This study found that alcohol abuse, transactional sex relations, sexually suggestive dressing, inadequate family protection, gender stereotypes, and poor legal literacy regarding sexual violence are key drivers of sexual violence. Apart from increasing the risk of sexual assault, the use of recreational drugs has been shown in literature to be a possible manifestation of deeper problems such as a significantly hostile growth environment or peer pressure. Hence, creating a vicious circle of sexual assault and drug abuse (29–31). Unfortunately, society tends to justify sexual violence when done under the influence of alcohol or drugs. Ironically, literature shows that girls and young women have a tendency not to report sexual assault, which occurs when either of the partners is under the influence of alcohol or drugs (32, 33). As a result, the relationship between alcohol and sexual assault should be considered when dealing with sexual assault.

Some AGYW get sexually assaulted as they try to get food, menstrual hygiene products, money, school materials, and other goods and services. By denying women meaningful economic and livelihood activities and limiting women's access to basic needs, AGYW from low-income families are prone to exploitation and sexual assault compared to their counterparts from wealthier households (12, 34). Available jobs for AGYW school dropouts are mostly in drinking establishments such as bars and local restaurants, where the customers see them as objects of sexual pleasure (31, 35). On the other hand, some societies believe that revealing clothes are indecent and may objectify AGYW and attract unnecessary attention from males, resulting in sexual assault (36, 37).

#### Inadequate family protection

It was worrying that some families could fail to report sexual assault and protect victims. The Rwandan DHS (12) and WHO (1) indicated that the highest number of women who were sexually abused were mostly with people close to or intimate with them. Being silent on such cases may promote sexual assault by family, relatives, neighbours, and friends as victims fear retaliation. In addition, parental protection was found to be lacking owing to school and work commitments. While parental protection positively mitigates sexual assault, it is becoming commonplace with changing global trends (38, 39). This may imply that more structures and resources should be considered for SRHR and the prevention of sexual violence since family time is becoming curtailed. Also, families may need to focus more on quality rather than quantity of family time.

#### Gender stereotypes and misconceptions

At the community level, patriarchal tendencies, such as male dominance, superiority, and control, were often pointed out by participants as restricting the agency and autonomy of women and girls and contributing to the normalisation of violence against AGYW. Patriarchy has been known to enable sexual violence (40, 41). There is a conflict between cultural norms and tradition, de-ethnicisation, and gender equality objectives that result in persistent violence against women and girls (42). These practices tend to give men control over women's and girls’ bodies and sexuality, resulting in far-reaching consequences such as harassment, sexual abuse, and rape. Therefore, it is important to challenge and transform patriarchal influences that contribute to economic and power imbalances, as well as all harmful cultural practices and gender stereotypes.

#### Poor legal literacy

At the societal level, this study found that many AGYWs do not know the fundamental laws protecting them against sexual violence. For instance, their rights, what constitutes violence, available support systems, and avenues for seeking justice. It is widely acknowledged in the literature that the lack of knowledge and ignorance surrounding sexual violence contributes to its perpetuation (12, 43, 44). Studies among school-going AGYW revealed that students who understood sexual violence as only involving rape were at higher risk of being sexually abused than those who understood all forms of sexual violence (44, 45). This indicates how limited knowledge of sexual violence intersects with other factors like poverty and makes them more vulnerable to sexual violence. Comprehensive sexuality and rights education may address this gap. These drivers, in line with feminist theory, tend to weaken AGYW, expose them to sexual violence, reduce chances for litigation and blame the women through entrenched gender stereotypes such as patriarchy and victim-blaming attitudes on how the AGYW dress or behave. Again, this shows that programs to prevent sexual abuse among AGYW should include empowerment initiatives, support, confidence building and addressing gender-based stereotypes.

### Optimising local violence mitigating resources

The study found some inherent resources which, if strengthened and applied, may reduce sexual violence against AGYW. These included parental guidance and *Ikirambi* intimate discussions, protective laws, public awareness and addressing of alcohol and drug abuse. Parental support and guidance (46, 47) have been shown to reduce the prevalence of sexual assault through protective guidance and enhanced security. Support from both parents is more effective than support from one parent, mainly if the parents can freely discuss sexual issues with the AGYW (48, 49). The *Ikirambi* practice offers opportunities for genuine intimate dialogues involving gender norms, sexual violence, and women's rights in safe environments. However, to be more effective, the *Ikirambi* may need deliberate linkages with other stakeholders so that the dialogues may lead to tangible outcomes and long-term solutions for addressing sexual violence. Such support and discussion prepare AGYW to recognise, assert boundaries and speak up, thereby preventing or addressing sexual violence. Other structural bottlenecks in addressing sexual violence may also need to be optimised. On the other hand, the need to address alcohol and drug abuse to curtail sexual violence has also been reported in literature (40, 50). Providing opportunities for women in the formal economy may also improve the autonomy of AGYW and reduce structural violence, as corroborated by literature (3, 51, 52). Lastly, the Rwandan laws are already deterrents for perpetrators of sexual violence. When consistently followed, the laws may reduce incidents of sexual violence (27, 53). The feminist perspective supports the need for comprehensive approaches to address sexual violence. Parental guidance, protective laws, increasing community awareness or addressing substance abuse all create safe and equitable environments for vulnerable groups such as AGYW to thrive (54). Hence, the importance of multifaceted interventions and societal consciousness in effectively addressing sexual violence and establishing a safer environment for everyone, especially AGYW.

#### Interference with judicial processes

This study found that families may conceal incidents of sexual violence. In some cases, corruption and bureaucracy may also stall judicial processes for sexual assault. Out-of-court settlements of criminal offences are still common. For convenience, safeguarding the dignity of offenders and societal ties, some communities are settling sexual violence cases through traditional informal agreements (55). However, such task-shifting of sexual violence recourse not only disadvantages girls and women in society but also neglects them and normalises the abuses that they experience. On the other hand, the police may also flaw incarceration and conviction through corruption and poor support of sexual assault survivors (56, 57). Hence, there is a need to deal with corruption and economically support survivors through judiciary processes. This aligns with feminism, which supports strategies that create equitable environments to promote vulnerable groups such as AGYW.

The four themes in this study regarding the drivers for and strategies to reduce sexual violence can be used for the assessment, planning and evaluation of interventions to prevent sexual violence among AGYW. From the four themes that emerged, four recommendations became obvious. Firstly, AGYW should be empowered with knowledge, skills, and financial autonomy to protect them from recreational drugs and sexual violence. This may promote the reporting of concealed sexual assault. Secondly, programs to reduce sexual violence should also consider the integration of several resources, which include families, community leaders, peers, cultural practices such as the *Ikirambi,* and legal channels for dealing with sexual assault. Integration may help support AGYW who survived sexual assault and those who have not encountered it. Thirdly, the litigation process needs to be shortened without overt corrupt tendencies to foster confidence in the process and deter offenders. Furthermore, the public should be made aware of sexual violence, its forms, handling, and prevention, as this may address rooted misconceptions about the violence. Lastly, efforts to address sexual violence should involve individuals, interpersonal relationships, the community, and society.

### Study limitations

This study adhered to the standards for reporting qualitative research (SRQR) guidelines. However, there are several limitations to consider. Firstly, only one of the researchers was fluent in the vernacular language used during data collection. Although we conducted several debrief sessions with research assistants to ensure accuracy, some nuances in the data may have been lost during translation and interpretation. Secondly, the sample size was small and drawn from a single district, limiting the diversity of perspectives captured. Additionally, as is typical with qualitative research, the findings are not intended to be generalisable but rather to provide rich, contextual insights into the specific experiences of participants.

Despite these limitations, this study remains significant as it amplifies the voices of those directly affected by sexual violence, particularly AGYW, and incorporates their perspectives into the public debate on the drivers and mitigators of such violence. Including these voices is crucial for shaping informed and effective interventions tailored to the realities of those impacted.

## Conclusion

This study found that the SRHR mentorship program empowers AGYW. Drivers for violence were found to be tiered according to the ecological framework and include practices perceived to increase vulnerability to sexual violence, inadequate family protection, gender stereotypes and misconceptions and poor legal literacy among people. Other themes included optimising existing violence, mitigating resources, and preventing interference with the judicial system. The study serves as a foundation for designing effective programs and policies to prevent and address sexual violence, ultimately contributing to the existing literature on this critical public health issue. Efforts to address sexual violence should include AGYW, their families and peers, the community and society.

## Data Availability

The datasets presented in this article are not readily available because the data collected for this study consists of audio recordings and field notes. The study involved sensitive issues of sexual assault and there is a potential for identification of participants. Therefore, public sharing of the data is not feasible. However, summaries of the in-depth interviews and field notes may be availed to researchers who request them for valid, ethically sound reasons. Request of the data can be made through the UGHE IRB and the corresponding author. Requests to access the datasets should be directed to Chairman, irb@ughe.org.
